# Gut microbiota from metabolic disease-resistant, macrophage-specific RIP140 knockdown mice improves metabolic phenotype and gastrointestinal integrity

**DOI:** 10.1038/srep38599

**Published:** 2016-12-08

**Authors:** Yi-Wei Lin, Emmanuel Montassier, Dan Knights, Li-Na Wei

**Affiliations:** 1 Department of Pharmacology, University of Minnesota Medical School, Minneapolis, MN 55455, USA; 2 Department of Computer Science and Engineering, University of Minnesota, Minneapolis, Minnesota 55455, USA; 3 Université de Nantes, EA 3826 Thérapeutiques cliniques et expérimentales des infections. Faculté de médecine, 1 Rue G Veil, 44000 Nantes, France; 4 Biotechnology Institute, University of Minnesota, Saint Paul, Minnesota, 55108, USA

## Abstract

While fecal microbiota transplantation (FMT) presents an attractive therapeutic strategy, it remains unclear how to choose the microbiota repertoire that most effectively transfers benefit to recipients. We identified a beneficial taxonomic repertoire in a transgenic mouse model (RIP140mϕKD) which resists the development of high fat diet (HFD)-induced metabolic diseases due to enhanced anti-inflammation engineered by lowering receptor interacting protein (RIP140) expression in macrophage. We confirmed using FMT from HFD-fed RIP140mϕKD to wild type (WT) mice that recipient mice acquired the microbiota repertoire of donor mice. Importantly, FMT from RIP140mϕKD to WT not only effectively transferred the beneficial taxonomic repertoire to WT recipients, but also enabled recipient animals acquiring the anti-inflammatory status of RIP140mϕKD donor animals and avoid HFD-induced insulin resistance, which is associated with significantly improved intestinal integrity. We conclude that FMT can transfer not only microbiota but also the donors’ intestinal innate immune status and improved intestinal integrity.

Metabolic syndrome, including obesity, high blood pressure, hyperglycemia, high levels of serum triglyceride level, and low level of high-density lipoprotein (HDL), presents a serious health condition associated with metabolic diseases such as type 2 diabetes and cardiovascular diseases^1^. Recent studies have demonstrated that intestinal homeostasis, harmonious interactions between the microbiota and the host immune system, is critical to the maintenance of normal physiology, metabolism and immune regulations^2^,^3^, and that the gut microbiota are involved in the development of numerous diseases^4^. The diversity and composition of intestinal microbiota could be altered by the host’s diet and/or genome, and is associated with the host’s systemic and local inflammatory responses that are critically important in the development of metabolic diseases^4^,^5^,^6^,^7^. Manipulation of the gut microbiota by fecal microbiota transplantation (FMT) has been applied in various disease models and clinical trials, such as inflammatory bowel diseases (IBD), *Clostridium difficile* infection (CDI) and neurodevelopmental disorders^8^,^9^,^10^,^11^; however, the specific taxonomic repertoire for protection, particularly under a disease-prone condition such as high fat diet (HFD) feeding, has never been identified. Further, the underlying mechanism remains elusive.

Receptor interacting protein 140 (RIP140, also known as NRIP1) is a wide-spectrum nuclear coregulator involved in various biological processes leading to diseases^12^,^13^. Specifically related to metabolic regulation where inflammation plays a key role, RIP140 is a critical regulator of innate immunity, mediated by its functions in macrophage polarization. RIP140 is a M1 activator by co-activating NF-κB^14^, and a M2 repressor through suppressing STAT6^15^. We previously generated mice with macrophage-specific knockdown of RIP140 (RIP140mϕKD)^14^ where the M2 macrophage population is dramatically expanded and the M1 macrophage population is reduced. These mice are protected against endotoxin shock^14^ and HFD-induced metabolic diseases^16^,^17^, and exhibit improved wound healing^15^. Further, their local innate immunity, such as anti-inflammatory potential in adipose tissues, is dramatically improved^17^,^18^. We therefore speculated possible beneficial shifts in their gut microbiota and intestinal innate immune status, and tested the potential to transfer, via FMT, their protective phenotype to recipient wild type (WT) animals also under HFD feeding.

We first determined the repertoire of intestinal microbiota affected by genotype, i.e., RIP140mϕKD mice vs. WT mice, and then carried out FMT, reciprocally, to validate efficacy of the transfer of gut microbiota. We found that FMT, from RIP140mϕKD to WT, transferred not only the specific taxonomic repertoire of RIP140mϕKD to WT recipient but also their intestinal innate immune feature (elevated M2 anti-inflammatory population) and tissue integrity. Both of these transferred features are associated with protection against HFD-induced metabolic abnormality in recipient animals. This study defines a specific taxonomic repertoire that confers protection against diet-induced metabolic diseases, and validates the efficacy of FMT in treating metabolic syndrome. The study also suggests a protective mechanism via transferring microbiome-associated intestinal innate immunity and tissue integrity.

## Results

### Genotype alters the composition and functional repertoire of intestinal microbiota

**Figure 1:**
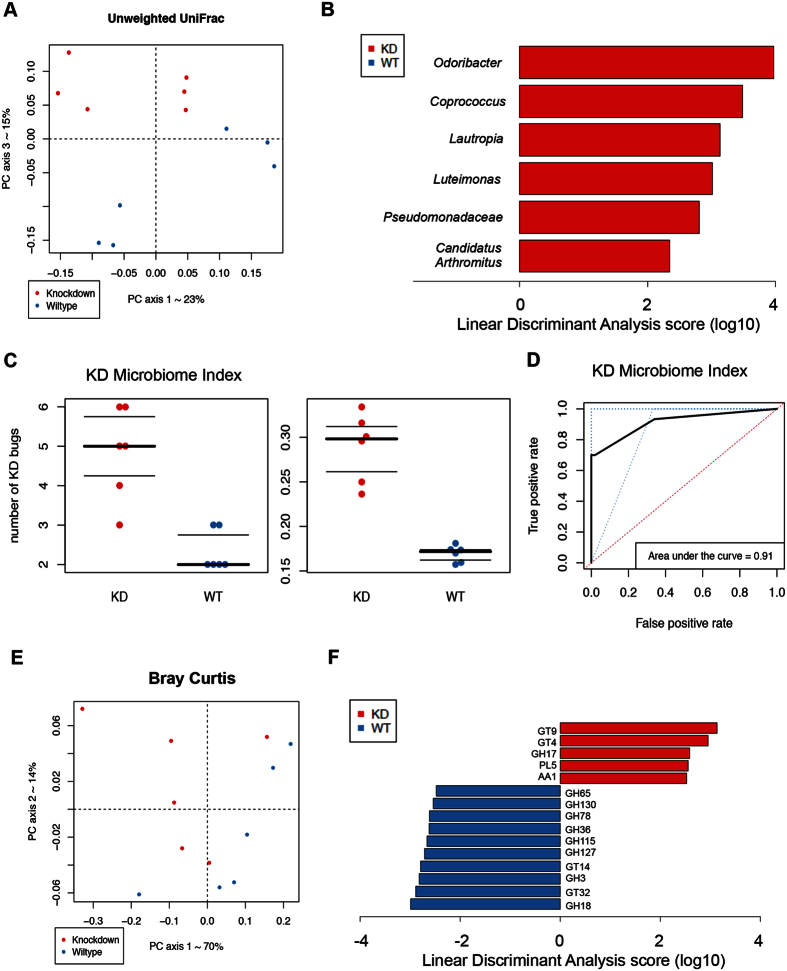
Macrophage RIP140 level alters the composition and functional repertoire of intestinal microbiota. (**A**) Beta diversity comparisons of the gut microbiomes of the fecal samples collected from WT and RIP140mϕKD mice receiving High Fat Diet. Analyses were performed on 16 S rRNA V4 regions data with a rarefaction depth of 66677 reads per sample. Principal coordinates analysis of Unweighted UniFrac distances. Proportion of variance explained by each principal coordinate axis is denoted in the corresponding axis label. The plot shows a separation between samples from WT and RIP140mϕKD mice receiving High Fat Diet (PERMANOVA, p = 0.04). (**B**) Summary of the taxa that differentiate WT from RIP140mϕKD mice receiving HFD using Linear discriminant analysis Effect Size analysis (LEfSe). (**C**) Left: KD microbiome index corresponding to the sum of number of genera among the differentiating taxa. Data were presented with Mann–Whitney U test: p-value = 0.007. Right: KD microbiome index corresponding to the total relative abundance of the differentiating taxa. Data were presented with Mann–Whitney U test: p-value = 0.002. (**D**) ROC curve analysis for KD microbiome index. (**E**) Beta-diversity plots from Bray-Curtis distance matrices for genome analysis. (**F**) CAZY GH assignments for glycoside hydrolase families analysis.

We first confirmed that HFD affected the composition of the gut microbiota (Fig. S1A). Interestingly, when comparing the effect of genotype, we found that the effect was most profound under HFD feeding, based on 16 s rRNA sequences of unweighted UniFrac distance metric (Fig. 1A, S1B and S1C) (PERMANOVA, p = 0.04). Moreover, supervised learning using Random Forests, a machine learning method using OTUs as predictive features, accurately assigned samples to their source population based on taxonomic profiles at the OTU level (83.3% accuracy, 3 times better than the baseline error rate for random guessing).

Using LEfSe^19^, we found that 6 genera were significantly different between WT and RIP140mϕKD mice fed a HFD (LDA log10 score >2). Specifically, RIP140mϕKD profile was associated with a significant gain in *Odoribacter, Coprococcus, Lautropia, Luteimonas, Candidatus Arthromitus and Pseudomonadaceae* when compared to WT mice. (Fig. 1B, S1D). We then constructed a KD microbiome index from this panel of taxa that highly differentiated between WT and RIP140mϕKD mice under HFD. This KD index corresponded to the sum of relative abundances of the 6 differentiating taxa. We found that the median KD index was 0.30 (IQR = 0.05) in RIP140mϕKD mice and 0.17 (IQR = 0.01) in WT mice (Mann-Whitney U test, p = 0.002) (Fig. 1C, right panel). Moreover, ROC curve analysis showed that our KD index was a strong predictor of the genotype, with an area under the curve of 0.91 (Fig. 1D). In order to determine the KD index threshold that best predicts genotype, we performed leave-one-out cross-validation on our KD indices. Each held-out KD index was treated as a new sample, independently from the initial cohort, on whom we tested and subsequently refined the optimal index cutoff to separate WT and RIP140mϕKD. This Leave-one-out (LOO) cross-validation procedure demonstrated that the taxon panel was able to predict the genotype in a new sample, with an accuracy of 83% at a specificity of 80%. Thus, our LOO analysis predicted genotype with reasonable accuracy and identified taxa associated with resistance to diet induced metabolic diseases that can serve as future biomarkers. These data demonstrated that genotype, that is WT vs. RIP140mϕKD profile, is strongly associated with a specific taxonomic repertoire in mice fed a HFD and that only 6 taxa can be used to predict the genotype of a mouse

Using PICRUSt^20^, we found that the gut microbiota functional repertoire is significantly different between WT and RIP140mϕKD mice. This algorithm estimates the functional potential of microbial communities given the current 16 S rRNA gene survey and a set of currently sequenced reference genomes. PICRUSt predictions in human and mouse gut microbiomes are expected to have 80–90% accuracy. First, beta-diversity plots generated from Bray–Curtis distance matrices showed a separation between WT and RIP140mϕKD mice fed a HFD (PERMANOVA, p = 0.041) (Fig. 1E). Moreover, supervised learning using Random Forests, with the predicted metagenome table collapsed at level 3 KEGG Orthology groups as predictive features, accurately assigned samples to their source population based on predicted metagenomic profiles (75% accuracy, 2 times better than the baseline error rate for random guessing). We also identified significant differences in microbial functional pathways in the fecal samples of WT and RIP140mϕKD mice fed a HFD (level 3 KEGG Orthology groups, Mann-Whitney U test, False Discovery Risk corrected p-value <0.20). The fecal microbiome of RIP140mϕKD mice is enriched in functional categories associated with fatty acid metabolism, lysine, valine, leucine and isoleucine degradation, caprolactam degradation, styrene and atrazine degradation, and depleted in categories associated with galactose metabolism, purine metabolism, cysteine and methionine metabolism, D-Glutamine and D-glutamate metabolism, nicotinate and nicotinamide metabolism, amino sugar and nucleotide sugar, thiamine metabolism and primary bile acid biosynthesis (Table S1).

Using CAZY GH assignments, we found that 5 glycoside hydrolase families were increased in RIP140mϕKD mice as compared to WT mice, including GH17, PL5, GT4, GT9 and AA1, whereas 10 glycoside hydrolase families showed a loss of abundance in KD mice as compared to WT mice, including GT14, GH36, GH3, GH18, GH115, GH78, GH65, GH130, GT32 and GH127 (LDA score (log10) >2) (Fig. 1F)

Together, these findings demonstrate that, in addition to a change in taxonomy, the genotype is associated with marked change in functional profile and glycoside hydrolase repertoire in animals fed a HFD.

### Fecal microbiota transplantation transfers gut microbiota from donor to recipient mice

**Figure 2:**
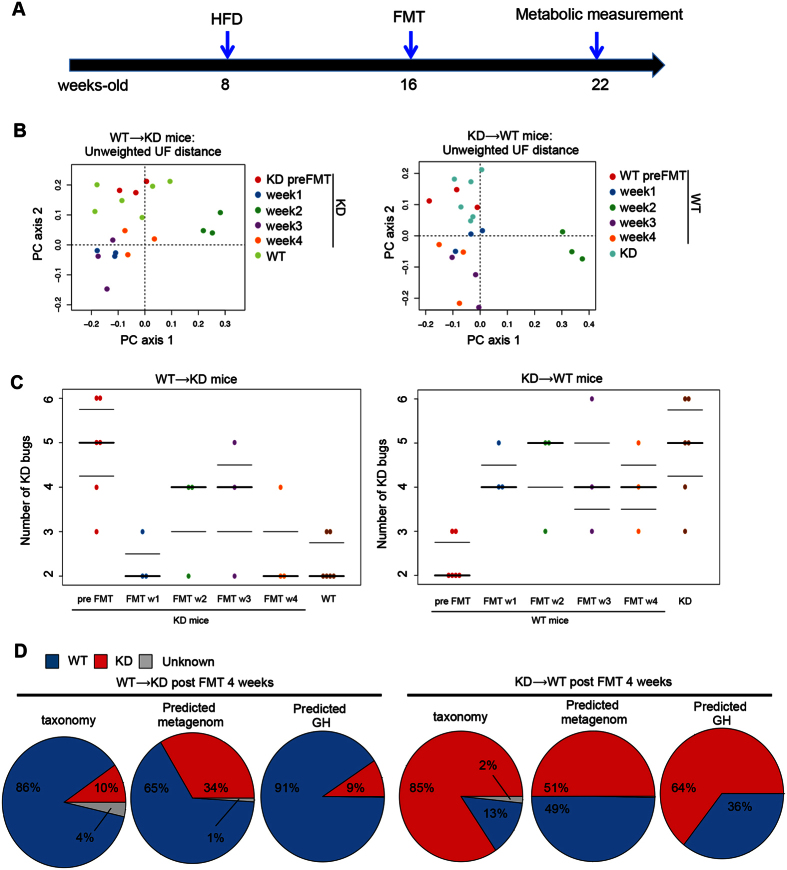
FMT transfers gut microbiota from donor to recipient. (**A**) A scheme showing FMT experiment. (**B**) Left: Unweighted UniFrac based PCoA from RIP140mϕKD mice receiving FMT from WT (WT → KD) mice. Right: Unweighted UniFrac based PCoA from WT mice receiving FMT from RIP140mϕKD (KD → WT) mice. (**C**) Left panel: KD microbiome index in RIP140mϕKD mice receiving FMT from WT (WT → KD) mice. Right panel: KD microbiome index in WT mice receiving FMT from RIP140mϕKD (KD → WT) mice. (**D**) Representative pie chart of Bayesian source-tracking analysis of taxonomy, predicted metagenome and predicted GH of WT → KD mice post FMT 4 weeks (left panel) and KD → WT mice post FMT 4 weeks (right panel). Source contributions were averaged across samples within the population. WT → WT: WT receiving WT, KD → WT: WT receiving KD, WT → KD: KD receiving WT and KD → KD: KD receiving KD.

Our previous studies showed that RIP140mϕKD mice are resistant to diet-induced metabolic diseases^16^,^21^. Since gut microbiota are strongly associated with hosts’ health, we proposed that gut microbiota in RIP140mϕKD mice could be associated with their metabolic protection features particularly under HFD. We thus performed reciprocal FMTs, from WT or RIP140mϕKD fed a HFD into WT or RIP140mϕKD mice in four groups: WT → WT (WT receiving WT), KD → WT (WT receiving KD), WT → KD (KD receiving WT) and KD → KD (KD receiving KD). The experimental design is depicted in Fig. 2A.

To first validate FMT efficiency, we examined changes in the diversity of recipient KD mice receiving FMT from donor WT mice (WT → KD). Unweighted UniFrac based PCoA showed differences between fecal samples collected from recipient RIP140mϕKD mice before FMT (i.e. native RIP140mϕKD mice) and four weeks after FMT (WT → KD) (PERMANOVA, p = 0.04), but did not show significant differences between fecal samples of WT → KD mice four weeks after FMT and native WT mice (PERMANOVA, p = 0.19). Moreover, fecal samples of recipient RIP140mϕKD mice after FMT (WT → KD) differed in terms of overall diversity when comparing to fecal samples of native RIP140mϕKD mice (PERMANOVA, p = p 0.048), but did not differ when comparing to fecal samples of native WT mice (PERMANOVA, p = 0.192) (Fig. 2B, left panel). We also examined changes in diversity of recipient WT mice receiving FMT from donor RIP140mϕKD mice (KD → WT). Unweighted UniFrac PCoA showed differences between fecal samples of recipient WT mice before FMT (i.e. native WT mice) and four weeks after FMT (KD → WT) (PERMANOVA, p = 0.02), but did not show significant differences between fecal samples of KD → WT mice and native RIP140mϕKD mice (PERMANOVA, p = 0.06). Moreover, fecal samples of recipient WT mice after FMT (KD → WT) differed in terms of diversity when comparing to fecal samples of native WT mice (PERMANOVA, p = p 0.043), but did not differ when comparing to fecal samples of native RIP140mϕKD mice (PERMANOVA, p = 0.071) (Fig. 2B, right panel).

Using the KD microbiome index described above, we found that WT → KD mice acquired a WT fecal microbiota signature; KD → WT mice acquired a KD fecal microbiota signature. The KD index differed between native RIP140ϕKD mice (i.e. fecal sample collected before FMT) and WT → KD mice post FMT (MWU test, p = 0.012) but the KD index did not differ between native WT mice and WT → KD mice post FMT (MWU test, p = 0.28) (Fig. 2C, left panel). Following the same trend, the KD index differed between native WT mice (i.e. fecal sample collected before FMT) and KD → WT mice post FMT (MWU test, p = 0.0018) but the KD index did not differ between native KD mice and KD → WT mice post FMT (MWU test, p = 0.28) (Fig. 2C, right panel).

To further explore this trend, we performed Bayesian source tracking on each of the post FMT fecal samples. This allows us to estimate the contribution of bacteria from the native WT mice, native RIP140mϕKD mice or from ‘unknown’ sources (one or more sources absent from the training data) in the post FMT samples. We found that the fecal samples of WT → KD mice were dominated by bacteria, predicted genes or CAZY GH of native WT mice (Fig. 2D, left panel); on the other hand, fecal samples of KD → WT mice were dominated by bacteria, predicted genes or CAZY GH of native RIP140mϕKD mice (Fig. 2D, right panel).

Based on these findings, we conclude that WT → KD mice post FMT acquired the microbiota signature of the WT genotype and KD → WT mice acquired that of the KD genotype.

### Healthy gut microbiota ameliorate diet-induced metabolic syndrome

**Figure 3:**
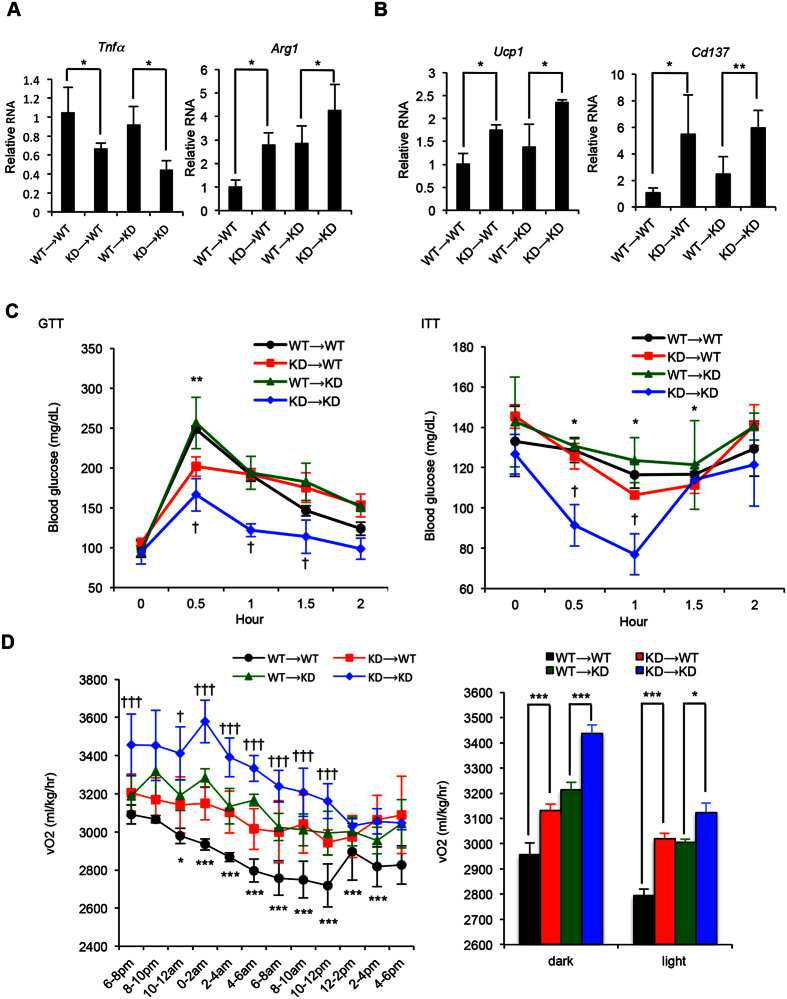
Receiving FMT from RIP140mϕKD mice ameliorates diet-induced diabetic traits. (**A**) qPCR of *Tnfa* (M1) and *Arg1* (M2) in visceral white adipose tissues. Student test (n = 3) was used and presented as mean ± SD, *P < 0.05. (**B**) qPCR of *Ucp1* (brown fat) and *Cd137* (beige fat) in visceral white adipose tissues. Student test (n = 3) was used and presented as mean ± SD, *P < 0.05; **P < 0.01. (**C**) GTT (left) and ITT (right) determined after 14 weeks of HFD feeding Student test (n = 3) was used and presented as mean ± SD, *P < 0.05, **P < 0.01 (WT → WT vs. KD → WT); ^†^P < 0.05 (WT → KD vs. KD → KD). (**D**) Energy expenditure of FMT mice. O_2_ consumption was measured in the dark and light phases and presented as vO_2_ (ml/kg/hr) (n = 3 in each group). Student test was used and presented as mean ± SEM. *P < 0.05; **P < 0.01; ***P < 0.001 (WT → WT vs. KD → WT), ^†^P < 0.05; ^††^P < 0.01; ^†††^P < 0.001 (WT → KD vs. KD → KD).

To determine if gut microbiota from RIP140mϕKD mice could benefit recipient mice under HFD, we performed a series of metabolic tests on post-FMT mice. RIP140mϕKD typically have elevated anti-inflammatory activities in their adipose tissues^17^,^22^. We therefore first examined if adipose innate immunity was affected by FMT. It appeared that mice receiving FMT from RIP140mϕKD mice (KD → WT) indeed have reduced M1 inflammatory marker (*Tnfα*) and elevated level of M2 anti-inflammatory marker (*Arg1*) in white adipose tissue (Fig. 3A). Furthermore, these mice express stronger beige (*Cd137*) and brown (*Ucp1*) fat markers (Fig. 3B). Mice receiving FMT from KD mice (KD → WT or KD → KD) were resistant to HFD-induced weight gain and insulin resistance compared to mice receiving FMT from WT mice (WT → WT or WT → KD) (Fig. 3C, Fig. S2). KD → WT mice show a higher rate of energy expenditure (O_2_ consumption) in both the light and dark phases (Fig. 3D). Taken together, our data show that FMT could transfer not only the “good” gut microbiota, but also the HFD-resistant, protective phenotype of the donor, RIP140mϕKD.

**Figure 4:**
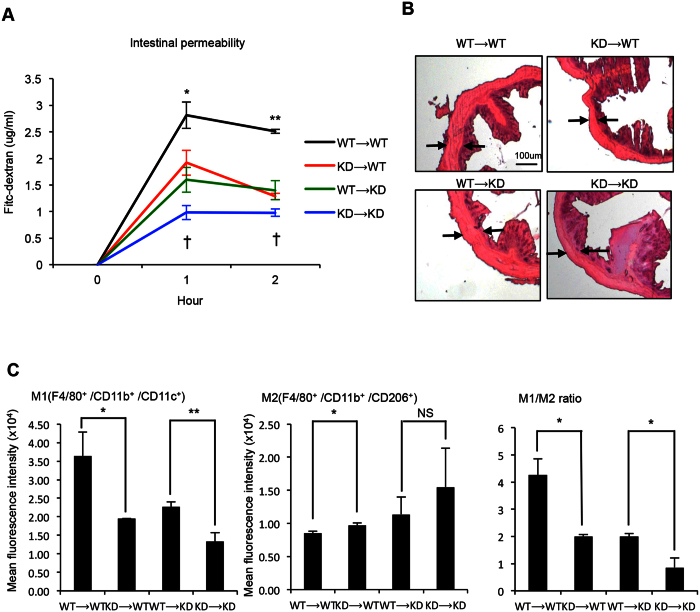
Receiving FMT from RIP140mϕKD mice improves colon health. (**A**) Serum levels of FITC-dextran in FMT mice. Student test was used and presented as mean ± SD. *P < 0.05; **P < 0.01 for comparison of WT → WT mice with KD → WT mice. ^†^P < 0.05 for comparison of WT → WT mice with KD → WT mice. (n = 3 in each group). (**B**) Representative H&E staining of colon sections. (**C**) FACS analysis of gut macrophage population in colon. Student test was used and presented as mean ± SD. *P < 0.05; **P < 0.01. (n = 3 in each group).

To gain insights into the possible mechanism, we examined the innate immune potential and tissue integrity of the gastrointestinal (GI) tract, because gut microbiota make direct contact with GI tract, affecting the microenvironment. We first monitored intestinal permeability (Fig. 4A) and found that both KD → WT and KD → KD mice have decreased intestinal permeability, indicating that they are less susceptible to low-grade inflammation that could contribute to the development of metabolic syndrome^23^. These mice also exhibit fewer pathological features in the colon, with apparently decreased hyperplasia (Fig. 4B). In terms of local (intestinal) innate immunity as monitored by M1 vs. M2 ratio (Fig. 4C), these mice have elevated M2 and lowered M1 markers and a reduced M1/M2 ratio indicative of improved anti-inflammation in the GI tract.

## Discussion

Our study is the first to identify microbiota-associated biomarkers that correlate with protection against diet-induced metabolic syndrome. The study also uncovers a specific taxonomic repertoire in mice with an elevated anti-inflammatory potential under HFD feeding, and validates the efficacy of FMT to transfer protection against HFD-induced metabolic diseases. The study also elucidates the mechanism involving augmentation in GI innate immune status and its tissue integrity.

Several taxa associated with RIP140mϕKD mice were previously found protective against insulin resistance or dyslipidemia. Thus our findings of specific intestinal microbiota enriched in RIP140mϕKD mice are consistent with a proposed role in glucose homeostasis for these commensal bacteria. In the present study, we found the specific taxonomic repertoire consists of 6 genera of bacteria enriched in RIP140mϕKD mice, which are correlated with resistance to diet-induced metabolic syndrome: *Odoribacter, Coprococcus, Lautropia, Luteimonas, Candidatus Arthromitus and Pseudomonadaceae. Odoribacter splanchnicus* was previously reported to be associated with a healthy fasting serum lipid profile, defined as a positive correlation with HDL cholesterol and a negative correlation with total- or LDL cholesterol. Importantly, *Odoribacter splanchnicus* was also positively correlated with HbA1c^24^. Another study reported that eradicating *Candidatus arthromitus* in the mouse gut microbiota resulted in metabolic changes that led to obesity^25^. A study also showed that non-caloric artificial sweeteners drove the development of glucose intolerance through induction of compositional and functional alterations to the intestinal microbiota including a decrease in *Candidatus arthromitus*^26^. Moreover, a study in humans reported a substantial reduction in abundance of *Coprococcus* in obese and nonalcoholic steatohepatitis (NASH) subjects as compared to healthy subjects^27^. Furthermore, Pseudomonadaceae was found increased in zebrafish larvae following probiotic supplementation that resulted in a down-regulation of the expression of genes that decrease glucose levels in the blood^28^. Thus the presence of some taxa could be helpful in assessing risk of developing diet-induced metabolic syndrome.

In addition, we found suggestive evidence that microbiota-associated functional modules are significantly different between WT and RIP140mϕKD mice using PICRUSt. Importantly, several of the KEGG modules differentiating WT and RIP140mϕKD mice are implicated in lipid and glucose metabolism. Nicotinamide, found depleted in RIP140mϕKD, was previously reported to be involved in the development of type 2 diabetes, through an increase in reactive oxygen species, subsequent oxidative stress and insulin resistance^29^. Purine, found depleted in RIP140mϕKD mice, could modulate insulin sensitivity and was associated with the development of diabetic microvascular complication^30^. D-Glutamine and D-glutamate metabolism, found depleted in RIP140mϕKD mice, was found strongly associated with insulin resistance in a previous study in humans^31^. Other investigations have highlighted associations of branched-chain amino acids (i.e., isoleucine, leucine, and valine) with obesity, impaired glucose tolerance, and insulin resistance, which is consistent with our findings that isoleucine, leucine, and valine degradation pathways are enriched in RIP140mϕKD mice^32^,^33^,^34^. Valine, of which degradation module was found increased in RIP140mϕKD mice, was associated with insulin resistance^35^. Cysteine and methionine metabolism, found decreased in RIP140mϕKD mice, were also previously associated with obesity and insulin resistance. In mice, cysteine intake decreases glucose tolerance, with up-regulation of lipogenic and diabetogenic enzymes that induce fat gain^36^. In humans, increased plasma total cysteine was associated with obesity and insulin resistance^37^,^38^. A study in rats showed that long-term exposure to atrazine caused obesity in those fed a HFD, leading to diabetes. This is also consistent with our findings that styrene and atrazine degradation are enriched in RIP140mϕKD mice^39^. Furthermore, amino sugar and nucleotide sugar metabolism, found decreased in RIP140mϕKD mice, was reported to have a direct linkage with diabetes and metabolic syndrome^40^.

We also found that several glycoside hydrolase families are correlated with RIP140mϕKD mice and protection against diet-induced metabolic syndrome. GH18, found decreased in RIP140mϕKD mice was reported to be a biomarker for endothelial dysfunction, atherosclerosis, insulin resistance, and Type 2 Diabetes Mellitus^41^,^42^. Thus the presence of some microbiota-associated glycoside hydrolase families could also assess risk in developing diet-induced metabolic syndrome.

We also demonstrated here that FMT can be performed reciprocally and effectively. WT mice receiving FMT from RIP140mϕKD mice acquired the RIP140mϕKD phenotype, and RIP140mϕKD mice receiving FMT from WT mice acquired the WT phenotype. Using Bayesian source tracking^43^, we showed that the taxonomy and functional modules associated with RIP140mϕKD were transferred to WT mice, suggesting that commensal bacteria and microbiota-associated modules correlated with resistance to diet-induced metabolic syndrome are substantially transferred to the diseased animals and augment disease outcome. Mechanistically, we found that FMT effectively transferred the protective innate immune phenotype (enhanced M2, anti-inflammatory potential) of the donor animals to the recipients under HFD, which also is associated with the improved GI tissue integrity in the recipients (Fig. 4C). These results provide a proof-of-concept that innate immunity affects gut microbiota, and vise versa, and that the protection can be effectively transferred by FMT. How microbiota affects host innate immunity, either systemically or locally, remains to be examined.

A small, randomized, double-blind controlled study showed that FMT, using stool from lean donors, significantly improves insulin sensitivity in obese male individuals, with butyrate-producing intestinal bacteria increasing in intestinal samples^8^. Our results validate the therapeutic efficacy of FMT from animals resistant to diet-induced metabolic syndrome, particularly those with improved innate immunity.

## Methods

### Animals and FMT procedure

Male C57Bl/6 mice were purchased from the Jackson Laboratory and maintained in the animal facility of University of Minnesota on a 12-h light/dark photocycle. All animal studies were approved and conducted according to guidelines of the University of Minnesota Institutional Animal Care and Use Committee. RIP140 mϕKD transgenic mice were generated as described before^14^. For experiments in Fig. S1A, 18 WT and 18 RIP140 mϕKD mice were used. For FMT experiments, we adopted a commonly used FMT experimental design where three individually housed animals had been used per group^44^,^45^,^46^,^47^,^48^,^49^. The procedure is descried as following. Briefly, eight-week-old male mice (6 WT and 6 KD) were housed individually and fed a high fat diet containing 60% calories from fat and 345 mg cholesterol/kg (F3282; Bio-Serv, West Chester, PA). After 7-weeks HFD feeding, feces was collected and stored for microbiome analyses and FMT later (pre-FMT samples). Fresh ampicillin solution (0.5 g/L) was added in drinking water every 3 days for a week^50^. FMT was then performed using pre-FMT samples, daily for three days, starting from 8-weeks of HFD feeding (one week post antibiotics treatment). Fecal samples were then collected, one week after FMT, for four more weeks. Metabolic measurement (see later) were performed. Animals were then sacrificed for collecting blood, colons and visceral white adipose tissues. Detailed sequence analyses and statistical analyses were provided in supplemental information.

### 16 S rRNA gene amplification and sequencing

Fecal samples were kept frozen at −80 °C until they were processed. After fecal DNA isolation (MoBio, Carlsbad, CA fecal DNA kit), amplicons spanning the variable region 4 of bacterial 16 S rRNA were generated and sequenced using Illumina Mi-seq platform at the University of Minnesota Genomic Center, Twin Cities, MN. The 16 S rRNA sequencing data from the Illumina runs were trimmed and chimera filtered using Quantitative Insights Into Microbial Ecology (QIIME) 1.9.1^51^ with default parameters which include several quality filtering steps for sequence lengths, end-trimming, and minimum quality score. We performed operational taxonomic units (OTUs) assignment using ‘NINJA-OPS’ against the Greengenes 13.8 database as a ref. 52.

### Hematoxylin and Eosin (H&E) Staining

After mice were sacrificed, colon tissues were fixed, embedded in paraffin, sectioned and stained as described previously^17^. Images were taken and analyzed by Zeiss Axioplan 2 Upright Microscope in University Imaging Centers in U. of Minnesota. Hemotoxylin and eosin staining kit was purchased from Fisher Scientific.

### Metabolic Measurement

Indirect calorimetry was performed as described previously^17^ to measure volumetric oxygen consumption (vO2) on mice. Briefly, animals were housed in individual chambers with free access to food and water in a 12-h light/dark cycle. vO2 was recorded and normalized to body weight using Oxymax (Columbus Instrument, Columbus OH).

### Glucose tolerance test (GTT) and insulin tolerance test (ITT)

GTT and ITT were performed after overnight fasting. Animal baseline blood samples were collected. D–glucose (2 g/kg) or insulin (0.75 units/kg) was i.p. injected into animals. Blood glucose levels were measured at indicated time points with a glucometer (OneTouch Ultra).

### Gut permeability assay

*In vivo* gut permeability assay was performed as described previously^53^. Briefly, mice were fed 0.6 mg/g FITC-labeled dextran (Sigma) after fasting for 5 hrs. Serum samples were collected at indicated time points and analyzed (485 nm/535 nm) using a microplate reader (Tecan Infinite M100 pro).

### RNA isolation and quantitative real-time PCR

RNA was isolated using Trizol. Reverse transcription was performed using a High-Capacity cDNA Reverse Transcription Kit containing RNase Inhibitor (Applied Biosystems). Quantitative real-time PCR (qPCR) was carried out with Maxima SYBR Green qPCR Master Mixes (Thermo Scientific) as described previously^16^. Primers for *Tnfα* (QT00104006), *Arg1* (QT00134288), *Ucp1* (QT00097300) and *Cd137* (QT00147266) were purchased from Qiagen. Each analysis was performed triplicate and normalized to β-actin.

### Flow Cytometry

Fc Block (20 m g/mL; BD Biosciences) was used to block cell-surface antigens. After blocking cells were stained with fluorophore conjugated antibodies or isotype control antibodies. Fluorophore-conjugated primary antibodies were purchased from BioLegend: F4/80-Alexa Fluor 488, CD11b-PerCP/Cy5.5, CD11c-phycoerythrin, and CD206-Alexa Fluor 647. After incubation with antibodies, cells were washed and centrifuged, re-suspended in a washing buffer, and analyzed on a FACSCalibur using FlowJo 10.0.6.

## Additional Information

**How to cite this article**: Lin, Y.-W. *et al*. Gut microbiota from metabolic disease-resistant, macrophage-specific RIP140 knockdown mice improves metabolic phenotype and gastrointestinal integrity. *Sci. Rep.* **6**, 38599; doi: 10.1038/srep38599 (2016).

**Publisher’s note:** Springer Nature remains neutral with regard to jurisdictional claims in published maps and institutional affiliations.

## Supplementary Material

Supplementary Dataset
