# Supplementary material for: Gut microbiota from metabolic disease-resistant, macrophage-specific RIP140 knockdown mice improves metabolic phenotype and gastrointestinal integrity
Source: Sci Rep. 2016 Dec 8;6:38599. doi: 10.1038/srep38599 (PMC5144013; doi:10.1038/srep38599)
Supplement: Supplementary Dataset [file srep38599-s1.doc]

**Gut microbiota from metabolic disease-resistant, macrophage-specific RIP140 knockdown mice improves metabolic phenotype and gastrointestinal integrity**

Yi-Wei Lin^*, 1^, Emmanuel Montassier^*,2, 3^, Dan Knights^2,4^, and Li-Na Wei^#,1^

* These authors contributed equally to this work

# correspondence should be sent to

Li-Na Wei, Department of Pharmacology University of Minnesota Medical School, 6-120 Jackson 321 Church St. SE, Minneapolis, MN 55455

Tel (612)6259402; email “weixx009@umn.edu”

1. Department of Pharmacology, University of Minnesota Medical School

2. Department of Computer Science and Engineering, University of Minnesota, Minneapolis, Minnesota 55455, USA

3. Université de Nantes, EA 3826 Thérapeutiques cliniques et expérimentales des infections. Faculté de médecine, 1 Rue G Veil, 44000 Nantes, France.

4. Biotechnology Institute, University of Minnesota, Saint Paul, Minnesota, 55108

**Figure S1. Taxonomic profile of the gut microbiome**

(A) Taxonomic profile of the gut microbiome of the fecal samples collected from WT and RIP140mφKD mice receiving ND and HFD. Relative taxa abundance plots summarized at family level. Mice are represented along the horizontal axis, and relative taxa frequency is denoted by the vertical axis. (N=9 in each group).

(B) Taxonomic profile of the gut microbiome of the fecal samples collected from WT and RIP140mφKD mice receiving HFD. Relative taxa abundance plots summarized at family level. Mice are represented along the horizontal axis, and relative taxa frequency is denoted by the vertical axis. (N=6 in each group).

(C) Beta diversity comparisons of the gut microbiomes of the fecal samples collected from wildtype and knockdown mice receiving High Fat Diet. Analyses were performed on 16S rRNA V4 regions data with a rarefaction depth of 66677 reads per sample. Principal coordinates analysis of Unweighted UniFrac distances with principal coordinates 1 and 2. Proportion of variance explained by each principal coordinate axis is denoted in the corresponding axis label. This plot showed a separation between samples from WT and RIP140mfKD mice receiving High Fat Diet (PERMANOVA, p = 0.04).

(D) Relative abundance of the taxa that differentiate WT and RIP140mφKD mice receiving HFD using Linear discriminant analysis Effect Size analysis (LEfSe).

**Table S1. Gut microbiome metagenome prediction**

Gut microbiome metagenome prediction. Black color represents genes increased in WT mice. Red color represents genes increased in RIP140mφKD mice.

**Figure S2. Body weight of mice**

Body weight of mice after HFD before sacrifice. Student test was used and presented as mean±SD. *P < 0.05, n=3 in each group.

**Supplemental Methods**

**Sequences analysis and Statistics.** Beta-diversity analyses were performed using unweighted UniFrac ^1^ (16S rRNA), and Bray–Curtis (PICRUSt metagenome predictions) ^2^ distance metrics. Statistical analyses including PCoA, PERMANOVA tests, were performed using QIIME 1.9.1 and R. In detail, we computed beta diversity metrics and visualized with Principal Coordinate Analysis (PCoA). We then tested the differences between samples from WT and RIP140mφKD mice with the Permutational Multivariate Analysis of Variance Using Distance Matrices (PERMANOVA) method. Moreover, supervised machine learning (Random Forest) ^3^ and Bayesian source-tracking ^4^ were performed. Machine learning methods were applied using Random Forest classifiers with 10-fold cross-validation and 1,000 trees ^3^. Comparisons of relative abundance of taxa between WT and RIP140mφKD mice was performed using Linear discriminant analysis Effect Size (LEfSe), a non-parametric Mann-Whitney U (MWU) test applied to detect features with significant differential abundance, followed by a Linear Discriminant Analysis (LDA) to estimate the effect size of each differentially abundant feature. As proposed, a LDA score (log 10) > 2 was considered significant ^5^.

A KD microbiome index was built to differentiate WT and RIP140mφKD mice, based on taxonomy using the OTU table collapsed at genus level. The objective of this KD index was to accurately identify genotype based on the taxonomic profile of the mice with a reduced number of taxonomic features, that is the taxa that highly differentiate WT and RIP140mφKD. All the taxa with a LDA score (log 10) > 2 were included in the calculation of the KD index. In order to build the KD index, in each mouse the number of taxa increased in the RIP140mφKD mice (among the taxa found with a LDA score (log 10) > 2) was summed and the number of taxa increased in the WT mice (among the taxa found with a LDA score (log10)>2) was summed. Then the difference between these two sums was calculated, thereby obtaining a KD index. This procedure was repeated n (overall sample size) times to obtain a KD index for each patient in the cohort. Then the KD index obtained in WT and RIP140mφKD mice were compared using a Mann Whitney U test.

Receiver operating characteristic (ROC) curves were plotted and the areas under the curve (AUC) values were computed on a dataset containing 10 sets of predictions and corresponding labels obtained from 10-fold cross-validation using ROCR package in R. A KD index threshold was also determined that best predicted genotype (i.e. WT or RIP140mφKD), with a leave-one-out (LOO) cross-validation on the KD indexes obtained in each mouse. We built an ROC curve that included KD indexes from n-1 mice. We then determined the best cutoff associated with a given specificity (80%). This cutoff was used to predict the outcome of the held-out sample. Thus, each held-out sample was treated as a new sample on which the optimal KD index cutoff was tested to separate WT and RIP140mφKD mice. This procedure was repeated n times and we calculated the accuracy of the cutoff by summarizing the good prediction of the outcome of each held-out sample. Boxplots, beeswarms and two-dimensional PCoA plots were generated using R.

We used Phylogenetic Investigation of Communities by Reconstruction of Unobserved States (PICRUSt), a computational approach to predict the functional composition of a metagenome using marker gene data (16S rRNA gene) and a database of reference genomes ^2^. Moreover, in order to determine the nutritional niches, we also used Phylogenetic Investigation of Communities by Reconstruction of Unobserved States (PICRUSt) to predict glycoside hydrolase (CAZY GH) assignments. We applied PICRUSt with defaults parameters to normalize the OTU table by dividing each OTU by the known/predicted 16S copy number abundance. Then, we used the precalculated file containing the CAZY GH assignments, untitled cazy_13_5_precalculated.tab, and apply the predict_metagenomes.py script as recommended, specifying CAZY GH assignments in the type of prediction. The output file gave the CAZY GH assignment in each sample included in the OTU table.


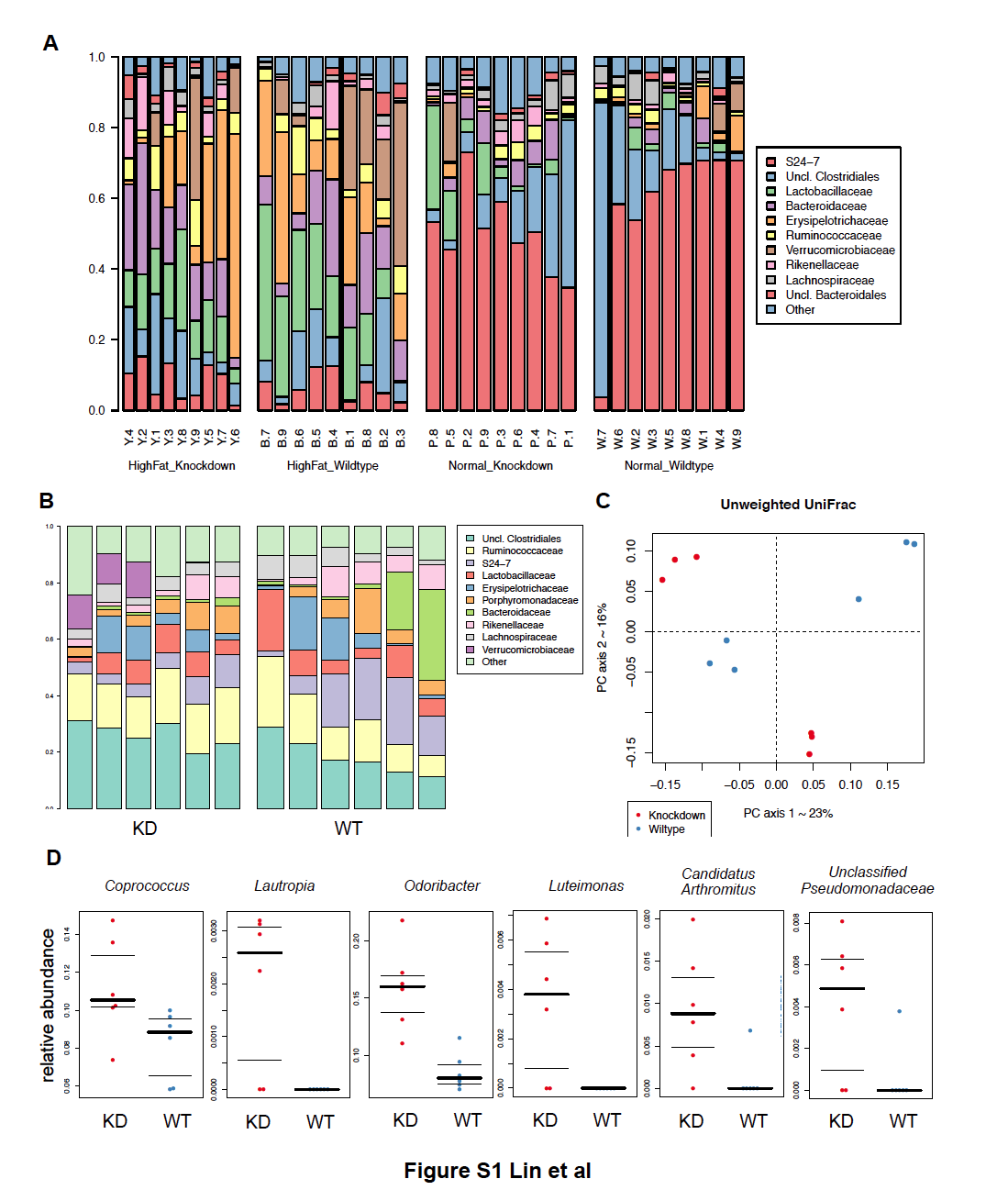


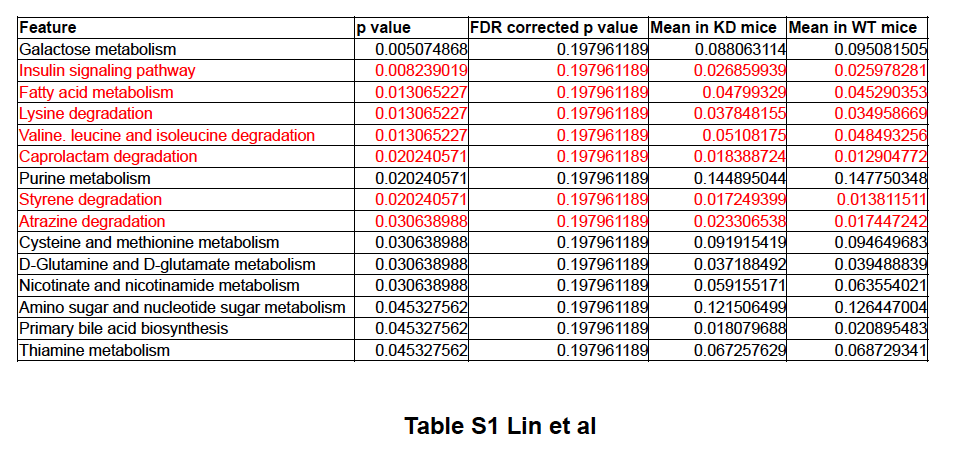


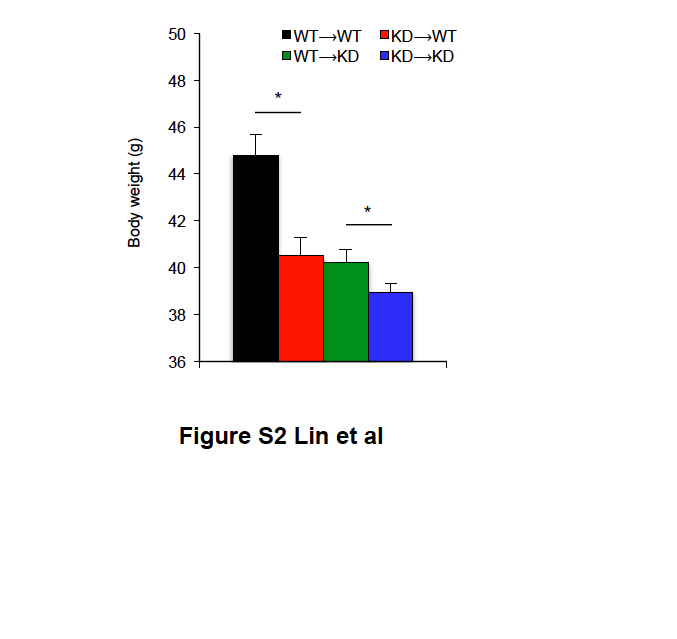


Reference:

1 Lozupone, C., Lladser, M. E., Knights, D., Stombaugh, J. & Knight, R. UniFrac: an effective distance metric for microbial community comparison. *The ISME journal* **5**, 169-172, doi:10.1038/ismej.2010.133 (2011).

2 Langille, M. G. *et al.* Predictive functional profiling of microbial communities using 16S rRNA marker gene sequences. *Nature biotechnology* **31**, 814-821, doi:10.1038/nbt.2676 (2013).

3 Knights, D., Costello, E. K. & Knight, R. Supervised classification of human microbiota. *FEMS microbiology reviews* **35**, 343-359, doi:10.1111/j.1574-6976.2010.00251.x (2011).

4 Knights, D. *et al.* Bayesian community-wide culture-independent microbial source tracking. *Nature methods* **8**, 761-763, doi:10.1038/nmeth.1650 (2011).

5 Segata, N. *et al.* Metagenomic biomarker discovery and explanation. *Genome biology* **12**, R60, doi:10.1186/gb-2011-12-6-r60 (2011).
